# Conceptualization, use, and outcomes associated with compassion in the care of people with multiple sclerosis: a scoping review

**DOI:** 10.1007/s00415-022-11497-x

**Published:** 2022-11-29

**Authors:** Robert Simpson, Stephanie Posa, Tania Bruno, Sharon Simpson, Marina B. Wasilewski, Lawrence R. Robinson, Sarah Munce, Mark Bayley, Anthony Feinstein

**Affiliations:** 1 Institute of Health and Wellbeing, University of Glasgow, Glasgow, UK; 2 Division of Physical Medicine and Rehabilitation, Temerty Faculty of Medicine, University of Toronto, Toronto, Canada; 3 St. John’s Rehab Research Program, Sunnybrook Research Institute, Toronto, Canada; 4 Rehabilitation Sciences Institute, University of Toronto, Toronto, Canada; 5 Division of Physical Medicine and Rehabilitation, Department of Medicine, Sunnybrook Health Sciences Centre, Toronto, Canada; 6 KITE Research Institute, University Health Network, Toronto, Canada; 7 University Health Network, Toronto Rehabilitation Institute, 347 Rumsey Rd, East York, Toronto, ON M4G 2V6 Canada; 8 Department of Psychiatry, Sunnybrook Health Sciences Centre, Toronto, Canada; 9 Consultation/Liaison Psychiatry, University of Toronto, Toronto, Canada

**Keywords:** Compassion, Multiple sclerosis, Scoping review

## Abstract

**Objective:**

Compassion is widely regarded as an important component of high-quality healthcare. However, its conceptualization, use, and associated outcomes in the care of people with multiple sclerosis (PwMS) have not been synthesized. The aim of this review is to scope the peer reviewed academic literature on the conceptualization, use, and outcomes associated with compassion in the care of PwMS.

**Methods:**

Studies were eligible for inclusion if reporting primary research data from quantitative, qualitative, or mixed-methods studies on the conceptualization, use, and outcomes associated with compassion in the care of PwMS. Relevant studies were identified through searching five electronic databases (CINAHL, Cochrane Library, EMBASE, MEDLINE, and PsycINFO) in January 2022. We followed the guidance outlined in the Joanna Briggs Institute (JBI) manual for evidence synthesis, and also referred to the Preferred Reporting Items for Systematic Reviews and Meta-analyses extension for Scoping Reviews Checklist (PRISMA-ScR). Simple descriptive methods were used to chart quantitative findings, and a descriptive approach with basic content analysis was employed to describe qualitative findings.

**Results:**

Fifteen studies were included (participant *n* = 1722): eight quantitative, six mixed-methods, one exclusively qualitative. Synthesized qualitative data revealed that PwMS conceptualize compassion as involving self-kindness, agency, and acceptance. PwMS report using self-compassion in response to unpleasant sensations and experiences. Quantitative findings suggest that compassion may mediate benefit finding, reduced distress, and improved quality of life (QoL) in PwMS, that those with the condition may become more compassionate through time, and that self-compassion specifically can be increased through training in mindfulness. In this context, greater self-compassion in PwMS correlates with less depression and fatigue, better resilience and QoL. Among studies, self-compassion was the most common outcome measure for PwMS.

**Conclusions:**

A nascent literature exists on the conceptualization, use, and outcomes associated with compassion in the care of PwMS. Further research is required to better understand what compassion means to PwMS and those caring for them. However, self-compassion can be cultivated among PwMS and may be helpful for managing unpleasant somatic symptoms and in benefit finding. Impact on other health outcomes is less clear. The use of compassion by health care providers in the care of PwMS is unstudied.

## Introduction

Multiple sclerosis (MS) is a chronic, progressive, neurodegenerative condition typically diagnosed between 20 and 40 years of age [1]. Around the world, incidence and prevalence of MS are increasing [2]. MS is an expensive condition, both for PwMS and for health services [3]. In the early stages, disease-modifying treatments are associated with most cost. As the condition progresses, social care costs dominate. However, around a third of all costs relate to intangible costs, deriving from patient suffering (stress, pain, fatigue), the so-called ‘hidden symptoms’ [4]. Indeed, people with multiple sclerosis (PwMS) commonly describe the condition as stressful, yet highlight how emotional aspects of care are frequently overlooked by their healthcare providers (HCPs) [5]. Stress is toxic for PwMS, increasing rates of anxiety and depression, and lowering quality of life (QoL) [6]. Unfortunately, effective mental health treatments for PwMS are limited, with current evidence favoring cognitive behavioral therapy (CBT) and mindfulness-based interventions (MBIs) [7, 8]. How CBT and MBIs improve stress, anxiety, and depression in PwMS is not entirely clear, though for MBIs compassion for oneself appears to play a mediatory role [9, 10]. Given the high prevalence of mental health impairment in PwMS, relative lack of effective treatments, and elevated care costs associated with hidden symptoms, it is important to explore novel treatments and self-management strategies that are acceptable, effective, affordable, and sustainable.

Compassion is a widely debated subject [11–13]. It has been defined empirically as the recognition of suffering in another coupled with a deep desire to alleviate that suffering [14]; and this latter aspect of compassion is suggested to differentiate it from empathy, which need not be coupled with a desire to alleviate suffering [15]. The empirical definition echoes earlier philosophical descriptions; Schopenhauer saw compassion as innate, being the basis for non-egoistic morality, justice and loving kindness [16]. From a biological perspective, compassion is thought to have evolved in mammals by necessity, to facilitate increased ‘in-group’ survival [17].

Compassion commonly features in mission statements and competency frameworks in many professional healthcare organizations [18–20], is regarded as an important component of quality healthcare [21], is widely taught in health professional education [22], and can be improved through targeted education [11, 23]. When patients perceive their HCP to be more empathic and compassionate, they report improved outcomes for stress, anxiety, depression, and pain [24]. Of concern, those medical specialists who routinely care for PwMS i.e., Neurology, Rehabilitation Medicine, and Primary Care providers are reported to have high levels of compassion fatigue [25] and burnout [26, 27]. which can lower empathic concern and compassionate responding through increased stress, emotional exhaustion, and depersonalization [28]

Both mindfulness- and compassion-based interventions can effectively reduce burnout and improve compassionate care in HCPs [29, 30]. Among patients (non-MS populations), interventions designed to cultivate compassion or self-compassion are associated with improvements in anxiety, depression, pain, and QoL [31, 32]. Indeed, in long term neurological conditions other than MS, greater self-compassion is correlated with improved resilience to stress, anxiety, and depression [33], though for unclear reasons effects are much smaller among those with chronic diseases, when compared to the general population [34].

To our knowledge, the academic literature on the conceptualization, use, and outcomes associated with compassion in the care of PwMS has not been synthesized previously, and the aim of this scoping review is to map the existing evidence in this area.

## Methods

The protocol for this scoping review was registered on the Open Science Framework Register on January 13, 2022, Registration https://doi.org/10.17605/OSF.IO/M5PHF. We used the Joanna Briggs Institute (JBI) Manual for scoping reviews [35] as a guiding framework, and referred to the Preferred Reporting Items for Systematic Reviews and Meta-analyses extension for Scoping Reviews Checklist (PRISMA-ScR) [36].

### Developing a search strategy

An initial search of the included databases was conducted, allowing analyses of text and index terms to be used across identified articles. Identified terms were integrated into our search strategy. Lastly, five databases were searched (CINAHL, Cochrane Library, EMBASE, MEDLINE, and PsycINFO), with medical subject headings and key words relating to compassion and MS, using controlled vocabulary, search symbols, and Boolean operators.

### Evidence screening and selection

#### Inclusion criteria

Studies were eligible for inclusion if reporting primary research data in English, from any year, inclusive of quantitative, qualitative, or mixed-methods findings on the conceptualization, use, and/or outcomes associated with compassion in the care of PwMS. If including other health conditions, data had to be extractable for PwMS specifically.

#### Screening and selection

We used Endnote and Covidence to store, screen, and sort results. Two reviewers (SP, RS) independently screened titles and abstracts of bibliographic records derived from the search. After removing duplicates, two reviewers conducted title and abstract review. Pilot testing of source selectors was conducted by assessing a random sample of 25 titles/abstracts, where our research team screened these using our eligibility criteria. We undertook full screening only once our team achieved > 80% agreement. Our agreement was defined by Cohen’s Kappa, *κ* = 0.83.

### Data extraction

Included studies were charted by two independent reviewers using the JBI manual data extraction template [37]. First, we performed a pilot extraction, whereby we trialed the extraction template for 2–3 sources to ensure all relevant results were being extracted. The following data were extracted: author(s), publication year, country, study aims, study type, methodology/methods, population/sample size, intervention/comparator, outcomes, and findings relating to the conceptualization, use, and outcomes associated with compassion in the care of PwMS.

### Analysis

Simple descriptive methods were used to chart the quantitative data, and a descriptive approach with conventional content analysis was undertaken to describe qualitative data [38]. An assessment of study quality was deemed overly complex with little added value given the wide ranging nature of study designs; an acceptable approach within scoping review methodology [36].

## Results

**Fig. 1:**
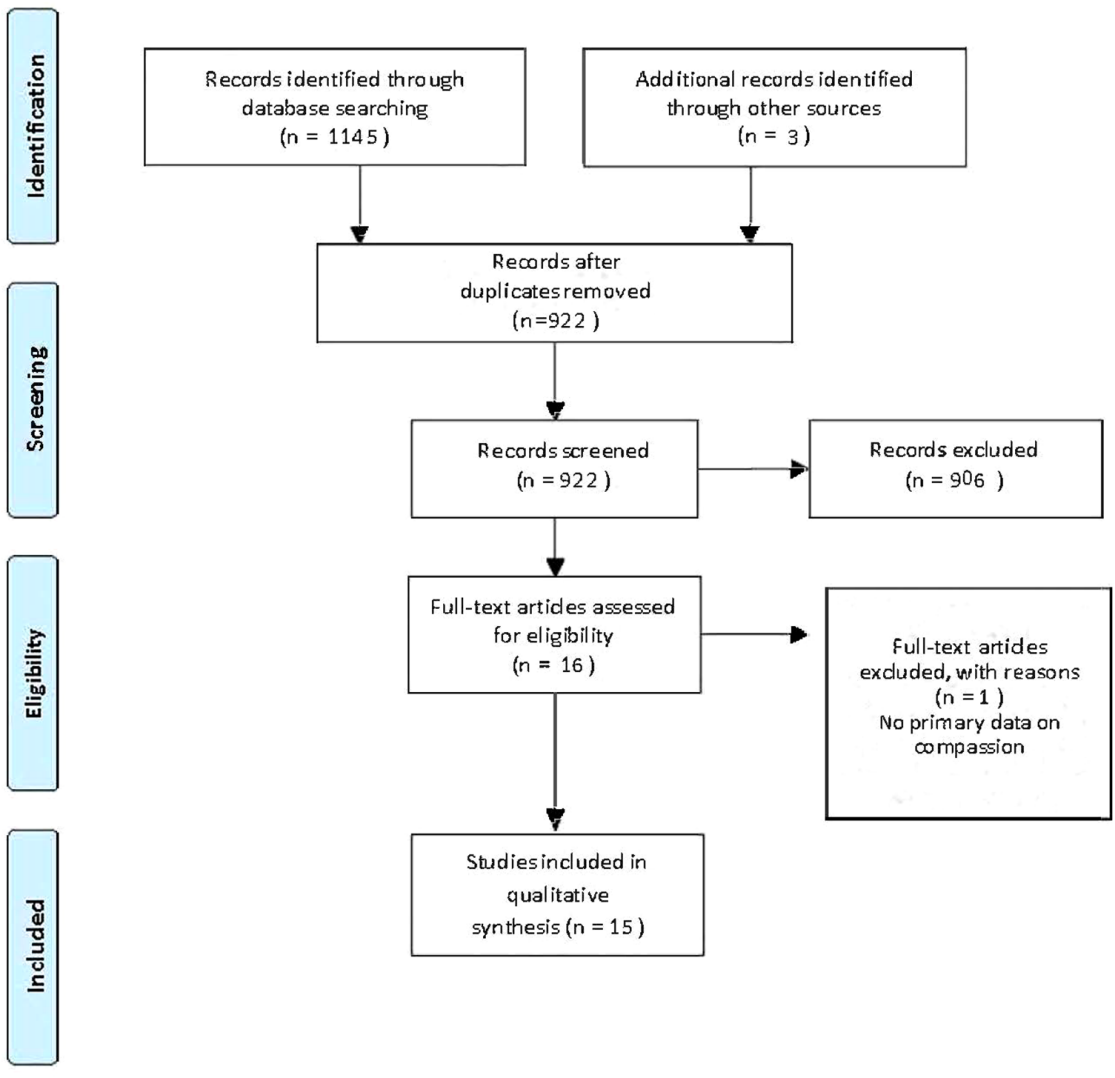
PRISMA flow diagram. Moher et al. [53]

Our search in January 2022 generated 1145 ‘hits’. Following de-duplication, there were 919 records. After title and abstract screening, 16 full text studies were deemed eligible, retrieved, and reviewed; however, one was a duplicate study. Thus, 15 articles were included in the final review. Search results are detailed in Fig. 1.

### Study characteristics

Eight studies used a quantitative methodology; one a randomized controlled trial (RCT) [39], four used cross-sectional surveys [40–43], one a longitudinal survey [44], one was a ‘unicentric prospective observational cohort study’ [45], and one a ‘cross sectional naturalistic design’ [46]. Six studies utilized a mixed-methods approach; one a ‘parallel pilot RCT and qualitative interview study’ [10], one a ‘pilot study’ with a single intervention condition [47], one described as a ‘quali-quantitative survey’ [48], one study comprised surveys and interviews [49], and two studies undertook respectively process and implementation analyses for an RCT [50, 51]. Lastly, one study adopted a qualitative approach using interviews [52]. Nine studies were conducted in Europe [10, 39, 41, 42, 45, 46, 48, 50, 51], two in Australia [44, 47], two in Asia [40, 52], one in the United States [43], and one between the United States and Europe (Table 1) [49].

**Table 1:** Study characteristics

Study	Setting/country	Study design	Population/n	Intervention	Intervention adherence	Outcomes	Data collection point(s)
Bogosian et al. [10]	Videoconference platform, United Kingdom	Mixed methods; quantitative data from a pilot RCT and qualitative interviews	PwMS, *n* = 40	MBCT	All participants attended = / > 4 of 8 MBI sessions. 14 (73.7%) attended = / > 6 sessions	GHQ-Total, AAQ-II, EQ, SCS-SF, SEMCD	Baseline, post-intervention, 3-months follow-up
Chesi et al. [48]	Italian MS centers, Italy	Mixed methods; quali-quantitative survey	Neurological teams for PwMS, *n* = 105	N/A	N/A	Satisfaction and Compassion Fatigue Test	Single time point
Dahmardeh et al. [40]	MS Associations, Iran	Quantitative; cross-sectional survey	PwMS, *n* = 51	N/A	N/ = A	RSES; Self-compassion researcher-made questionnaire	Single time point
Dahmardeh et al. [52]	Hospitals and communities, Iran	Qualitative; interviews	PwMS, *n* = 23	N/A	N/A	N/A	Single time point
Davidescu et al. [45]	Outpatient Neurology Department, Romania	Quantitative; unicentric prospective observational cohort study	PwMS, *n* = 122	N/A	N/A	DECAS Personality Inventory	Single time point
Gedik et al. [41]	Neurology Department of University Hospital, Turkey	Quantitative; descriptive and cross-sectional design	PwMS, *n* = 89	N/A	N/A	EDSS, MusiQoL, HADS, RSES, SCS	Single time point
Giménez-Llort et al. [46]	Specialized Health Service of Neurology, Spain	Quantitative; cross-sectional naturalistic design	PwMS and carers, *n* = 68	N/A	N/A	Quality of Life Scale, SF-36; SCS; CS; Modified FIS	Single time point
Ignatova et al. [42]	Hospital for Active Treatment-National Heart Hospital, hospital database, Bulgaria	Quantitative; cross-sectional survey	PwMS and healthy controls, *n* = 53	N/A	N/A	SCS, Reading the Mind in the Eyes Test, Faus Pas Recognition Test, ToM Cartoons	Single time point
Lex et al. [49]	Inpatient neuro rehab clinic and outpatient MS center, USA and Austria	Mixed methods; surveys and interviews	PwMS, *n* = 128	N/A	N/A	TPF, HAQUAMS, BFiMSS	Single time point
Nery-Hurwit et al. [43]	MS advocacy, support, exercise and education groups, USA	Quantitative; cross-sectional survey	PwMS, *n* = 259	N/A	N/A	CD-RISC10, FuNHRQOL-SF, SCS	Single time point
Pakenham et al. [44]	MS Society of Queensland, Australia	Quantitative; longitudinal survey	PwMS and carers, *n* = 620	N/A	N/A	MPAI, Marlow-Crown Social Desirability Scale, SOC-M, PSOM, Bradburn Affect Balance Scale, Symptoms Checklist-90, BFiMSS	Time 1, Time 2 (12 months later)
Simpson et al. [50]	NHS Centre for Integrative Care, Scotland	Mixed methods; process evaluation for an RCT	PwMS, *n* = 33	MBSR	6 interviewees did not complete course	N/A	Single time point
Simpson et al. [39]	NHS Centre for Integrative Care, Scotland	Quantitative; RCT	PwMS, *n* = 50	MBSR	60% attended = / > 4 MBSR sessions	PSS, EQ-5D-5L, MSQLI, MAAS, SCS-SF, ELQ	Baseline, post-intervention, 3 months later
Simpson et al. [51]	NHS, Scotland	Mixed methods; implementation analysis for an RCT	PwMS, MS clinicians and course instructors, *n* = 41	MBSR	Seven interviewees did not complete course	N/A	1 time point
Spitzer et al. [47]	Community based venues, Australia	Mixed methods; survey and interviews	PwMS, *n* = 23	MBCT	Of the 21 participants who completed pre- and post-intervention assessments, 80% (*n* = 17) attended four to five sessions, 15% (*n* = 3) attended three sessions, and 5% (*n* = 1) attended two sessions	Physician’s Disease Steps Scale; 13-item version of the Activities of Daily Living (ADL) Self-Care Scale for persons with MS; DASS-21; Perceived Stress Scale; SF-36; MFIS-5; SCS; AAQ-II; FFMQ-SF	Pre-intervention, post-intervention, follow-up

*PwMS* people with multiple sclerosis, *MBI* mindfulness-based intervention, *GHQ* General Health Questionnaire, *AAQ-II* Acceptance Action Questionnaire, *EQ* Experiences Questionnaire, *SCSSF* Self-Compassion Scale Short Form, *SEMCD* self-efficacy for managing chronic disease, *RSES* Rosenberg Self-Esteem Scale, *EDSS* Expanded Disability Status Scale, *MusiQoL* Multiple Sclerosis International Quality of Life Questionnaire, *HADS* Hospital Anxiety and Depression Scale; *SCS* Self-Compassion Scale, *TPF* Trierer Personlichkeitsfragebogen, *HAQUAMS* Hamburger QoL in Multiple Sclerosis Scale, *BFiMSS* Benefit Finding in Multiple Sclerosis Scale, *CD-RISC10* Revised Connor–Davidson Resilience Scale, *FuNHRQOL* Function, Neutral Health Related Quality of Life Short Form, *MPAI* Mayo-Portland Adaptability Inventory, *SOC-M* Sense of Coherence Scale, *PSOM* Positive States of Mind Scale, *MBSR* mindfulness-based stress reduction, *MBCT* mindfulness-based cognitive therapy, *RCT* randomized controlled trial, *PSS* Perceived Stress Scale-10, *EQ* EuroQoL, *MSQLI* Multiple Sclerosis Quality of Life Inventory; *MAAS* Mindful Attention Awareness Scale, *ELQ* Emotional Lability Questionnaire, *SF-36* Short Form Health Survey-36, *CS* Compassion Scale, *Modified FIS* Modified Fatigue Impact Scale

### Participant characteristics

The majority of included studies were comprised exclusively of PwMS [10, 39–41, 43, 47, 49, 50, 52]. Two studies also included healthy volunteer controls [42, 45] and another included PwMS and their ‘carers’ [44, 46]. Two studies featured MS clinicians [48, 51]. Disability was characterized according to the Expanded Disability Status Scale (EDSS) in eight studies [10, 39, 41, 42, 45, 49–51]. Of these, seven reported mean (SD) EDSS [10, 39, 41, 42, 45, 49, 51], which ranged from 1.51 (1.63) to 6.8 (1.6). One study reported an EDSS range of 1.0–7.0 [50]. Two studies reported ‘levels of severity of disease’ [40, 52], while two reported disability through the Activities of Daily Living Self-Care Scale for Persons with MS [44, 47]. Two studies did not measure disability, but rather general health [46] or health status [43]. In one study, participants were exclusively comprised of HCPs [48]. Participant ethnicity was reported in six studies [10, 39, 43, 45, 50, 51], with most participants being “White” or “Caucasian”. All but one study reported participant age [52], with most participants having an age range between 21 and 66, and median age between 40 and 50 across all studies. Participant sex was reported in all studies, with % female ranging between 57.3 and 94.0%. Four studies from Europe reported on socioeconomic status (SES); in one ‘middle class workers prevailed’ [45]; in another, 61% of participants perceived their income as ‘moderate’ [41]; another reported ‘postcode-derived SES of 4’ (on a scale of 1–10, 1 delineating the most deprived, 10 the least) [51] and a further study included participants ranging from the ‘most deprived’ to the ‘most affluent’ [50]. Education level was reported in 12 studies, with eight reporting that most participants completed higher education, university or college [10, 39, 41, 44–46, 50–52], three reporting that most participants had completed high school [40, 42, 49], and one reporting that most participants completed ‘TAFE/apprenticeship’ [47]. Across studies, the sample size ranged from 23 to 620, with a total of 1722 participants (Table 2).

**Table 2:** Participant characteristics

Study	Ethnicity	Number of participants/% female	Mean age (SD)	Socio-economic status	Employment status	Education status	Diagnoses
Bogosian et al. [10]	*Intervention:* 89.5% white British *Control:* 90.5% white British	Total *n* = 40 *Intervention:* 52.6% female *Control:* 61.9% female	*Intervention:* 53.42 (8.3) *Control:* 50.9(9.9)	N/R	N/R	*Intervention:* college or higher 68.4% *Control:* college or higher 85.7%	MS
Chesi et al. [48]	N/R	Total *n* = 105 69% female	46	N/R	*Neurologists* Permanent: 66% Fixed term: 17% Fellowship: 9% Freelance: 3% Internship: 1% Unspecified: 4% *Nurses* Permanent: 100%	N/R	N/A
Dahmardeh et al. [40]	N/R	Total *n* = 51 94% female	36.54	N/R	N/R	- Elementary school 5.8% - Guidance school 9.8% - Diploma 39% - Associate of science 15.6% - Bachelor’s degree 25% - Master’s degree 3.9%	MS
Dahmardeh et al. [52]	N/R	Total *n* = 23 86.9% female	N/R	N/R	N/R	- Illiterate 4.3% - High school 26% - Diploma 26%; - Bachelor’s degree 39%; - Master’s degree 4.3%	MS
Davidescu et al. [45]	“All Caucasians of Romanian origin”	Total *n* = 122 *Intervention* 71.31% female *Control* 71.31% female	42.06 (10.46)	“Middle class workers prevailed”	N/R	*Intervention* - 8 classes: 1.6% - 10 classes: 8.2% - Vocational school: 3.27% - High school: 31.14% - Post-secondary: 6.55% - University: 49.18% *Control* - 8 classes: 1.6% - 10 classes: 6.55% - Vocational school: 6.55% - High school: 34.42% - Post-secondary: 13.11% - University: 37.70%	MS
Gedik et al. [41]	N/R	Total *n* = 89	39.78 (10.83)	Measured through “perceived income” - Poor (5.6%); - Moderate (61.8%) - Good (31.5%) - Very good (1.1%)	- Employed (49.4%) - Unemployed (40.4%) - Retired (10.1%)	- Less than high school (25.8%) - High school (19.1%) - Bachelor’s degree (55.1%)	MS
Giménez-Llort et al. [46]	N/R	Total *n* = 68 *PwMS*: 70.45% female *Carers:* 66.67% female	*PwMS:* less than 36 (4.5%); between 36 and 45 (22.7%); between 46 and 55 (47%); > 55 (25%) *Carers:* less than 36 (29%); between 36 and 45 (8.3%); between 45 and 55 (37.5%); > 55 (25%)	N/R	N/R	*PwMS:* 50% had higher university studies, almost 30% studied until secondary school, 18.2% had completed primary studies, and only 2.3% had done no study *Carers:* Almost 80% had attended secondary school and/or university, and 20.8% had only primary education	MS
Ignatova et al. [42]	N/R	Total *n* = 53 *Patients with EDSS* < *3.5:* 72.2% female *Patients with EDSS* > *3.5:* 61.1% female *Control* 66.7% female	*Patients with EDSS* < *3.5:* 41.9 (11.6) *Patients with EDSS* > *3.5:* 43.7 (8.5) *Control:* 42.4 (12.3)	N/R	N/R	*EDSS* < *3.5:* 13.2 (2.3) years of education *EDSS* > *3.5:* 12.7 (1.7) years of education *Control:* 12.2 (3.5) years of education;	MS
Lex et al. [49]	N/R	Total *n* = 128 (64 from Austria and 64 from USA) 60.9% female	*Austria* 22.08 (8) *USA* 41.63 (8.64)	N/R	N/R	Did not complete secondary school (6.3%) - Completed secondary school (53.75%) - Completed higher education/university (40%)	MS
Nery-Hurwit et al. [43]	White (90.31%)	Total *n* = 259 84.17% female	48.55 (10.47)	N/R	Employed for wages: 38% Unable to work due to MS: 35.27%	N/R	MS
Pakenham et al. [44]	N/R	Total *n* = 620 82% female	49.33 (11.31)	N/R	Employed: 35% Pension or disability benefit: 38% Unemployed: 6% Retired: 20%	Primary school: 6% 10 years of education: 36% 12 years education: 17% University/trade education: 36%	MS
Simpson et al. [50]	All white Scottish	Total n *MBSR* = 50 Total *n* *interview* = 33 “Most were female”	Range 21–66	Ranged from most deprived deprivation level (decile 1) to the most affluent (decile 10)	N/R	Most had University education	MS
Simpson et al. [39]	100% white British	Total *n* = 50 *Control: *88% female *Intervention:* 92% female	*Intervention:* 43.6 (10.7) *Control:* 46.3 (11.1)	N/R	*Intervention:* Full time: (16%) Part time (12%) Unemployed (24%) Retired (20%) Other (28%) *Control:* Full time: (28%) Part time (24%) Retired (12%) Other (8%)	*Intervention:* Secondary school (12%) College (28%) University (60%) *Control:* Secondary school (20%) College (28%) University (52%)	MS
Simpson et al. [51]	100% “white Scottish ethnicity”	Total *n* = 41 88% female	44.3 (11.0)	A median SES of 4 (1 delineating the most deprived, 10 the least)	N/R	61% University level education	MS
Spitzer et al. [47]	N/R	Total *n* = 23 91.3% female	48.4 (9.6)	N/R	Full time: 30.4% Part time: 17.44% Voluntary work: 4.3% Retired: 8.7% Unable to work: 39.1%	High school: 21.7% TAFE/apprenticeship: 34.8% University bachelor: 17.4% Postgraduate: (26.1%)	MS

*N/A* not applicable, *N/R* not reported, *MS* multiple sclerosis, *EDSS* Expanded Disability Status Scale, *MBSR* mindfulness-based stress reduction, *SES* socio-economic status, *TAFE* technical and further education

### Intervention characteristics and adherence

Five studies were interventional [10, 39, 47, 50, 51], all being “Mindfulness-based”—with the aim being to decrease distress and improve psychological well-being. Each intervention occurred over 8 weeks, with sessions ranging from 1- to 2.5-h duration, and were facilitated by psychologists or physicians. One intervention ran virtually, over Skype [10], one was conducted across different community-based venues [47], and three were held in a medical clinic [39, 50, 51]. In the parallel pilot mixed-methods study from Europe, all participants completed four (or more) of eight sessions. In the pilot study from Australia, 80% of participants attended four to five of five sessions. In the RCT from Europe, 60% of participants met the criteria for course completion (> 4 sessions). Of the two mixed-methods studies conducted following a mindfulness-based stress reduction (MBSR) intervention, one reported that 17% of participants did not complete the course [51], while the other reported 18% attrition (Table 3) [50].

**Table 3:** Intervention characteristics

Study	Name of intervention	Intervention rationale	Intervention materials and procedures	Intervention providers	Intervention mode of delivery	Intervention frequency and duration	Tailoring or modifications	Adherence
Bogosian et al. [10]	Mindfulness-based cognitive therapy (MBCT)	To decrease distress in PwMS	Each group included 3–5 PwMS. Participants completed standardized questionnaires for the key outcomes and putative mediators, at baseline (pre-randomization), end of the intervention, and 3-month follow-up The wait-list groups were offered the mindfulness intervention at the final follow-up	A registered health psychologist, experienced with working with people with MS and a newly qualified mindfulness practitioner	Skype video	8-h long sessions; 8-week period	The MBCT sessions were tailored to address issues specific to people with MS	Eighteen of the 19 participants completed the MBCT. All the participants attended 4 or more of the 8 mindfulness sessions and 14 (73.7%) attended 6 or more sessions
Simpson et al. [39]	Mindfulness-based stress reduction (MBSR)	To assess effectiveness of intervention on outcome measures (perceived stress and QoL)	The intervention was based on standard MBSR, including home practice materials	Two physician facilitators with mindfulness teaching experience	In person	Weekly; 8 weeks	Excluded day retreat at week six for pragmatic, space-constraint reasons, as well as empirical evidence contesting its necessity	Fifteen participants (60%) attended four or more MBSR sessions, meeting the criteria for course ‘completion’. The average home practice time of 32.5 min per day was observed
Simpson et al. [50]	MBSR	To evaluate standard MBSR for people with MS in one group, then to optimize this based on participant feedback, and re-test it in a second group	MBSR is a complex intervention with three core treatment components (Mindful-breathing, mindful-body awareness, mindful-movement) The ‘standard’ MBSR course follows a protocol and consists of eight weekly sessions of 2.5 h, with additional ‘homework’ practice and a full-day silent retreat	Two MBSR instructors	In person	Weekly; 8 weeks	Modified MBSR course with modification based on findings derived from first group	MBSR session attendance rates (out of a possible eight) ranged from one to eight. Six interviewees had dropped out early in the course (after the first 1–2 sessions)
Simpson et al. [51]	MBSR	To examine barriers and facilitators to the implementation of an MBSR course for people with MS	Two successive groups of 25 people with MS receiving MBSR (total *n* = 50); the first group received standard MBSR; the second a version of MBSR with optimization changes based on feedback from group 1	Two MBSR instructors	In person	Weekly; 8 weeks	Rebranding of practices using culturally sensitive language. Mindful movement postures were simplified and adapted	MBSR session attendance rates (out of a possible eight) ranged from 1 to 8. There was a total of seven interviewees who had not completed the course
Spitzer et al. [47]	MBI	To improve QoL and psychological well-being among PwMS	In-session mindfulness followed by debriefing and discussion. Instruction on informal mindfulness skills, such as bringing mindful awareness to everyday activities, was integrated into each session. 30 min daily home practice encouraged. Participants unable to attend a session were contacted by the facilitator via 10–15 min phone-calls following the group session and given a brief session overview and the home practice	A registered psychologist	In-person community-based venues: a local library and a community disability service venue	2 h weekly sessions; 5 weeks	Mindful movement exercises were not included to enable people with mobility limitations to attend, regular breaks were used to accommodate MS fatigue, and self-compassion and acceptance instructions were incorporated into some exercises and group discussions	Of the 21 participants who completed pre- and post-intervention assessments, 80% (*n* = 17) attended four to five sessions, 15% (n = 3) attended three sessions, and 5% (*n* = 1) attended two sessions

*PwMS* people with multiple sclerosis, *MBI* mindfulness-based intervention, *QoL* quality of life, *MBCT* mindfulness-based cognitive therapy, *MBSR* mindfulness-based stress reduction

### Conceptualization of compassion in the care of PwMS

Compassion and compassion-related constructs were explicitly defined a priori in eight studies [10, 40, 41, 43, 46–48, 52]. Posteriori conceptualizations across studies solely spoke to the construct of self-compassion [10, 47, 50–52].

Among a priori definitions, self-compassion was defined most often (*n* = 7) [10, 40, 41, 43, 46, 47, 52], followed by compassion (*n* = 1) [46], and compassion fatigue (*n* = 1) [48]. Each construct was reflected in the process measures chosen within respective studies. Across studies, compassion was measured using a variety of validated outcome measures, including the Self-Compassion Scale-short form [10, 33], the Self-Compassion Scale [35–37, 40, 41], and the Compassion Scale [40]. One study utilized a non-validated ‘self-compassion researcher-made questionnaire’ [34]. Other studies used proxy measures for compassion, such as compassion satisfaction and compassion fatigue [48], the DECAS [Deschidere (Openness), Extraversiune (Extraversion), Conştiinciozitate (Conscientiousness), Agreabilitate (Agreeableness)] Personality Inventory [39], and the Benefit Finding in Multiple Sclerosis Scale (BFiMSS) [38, 43]. Other quantitative outcomes evaluated in relation to compassion included acceptance, QoL, distress, perceived stress, and self-esteem.

In qualitative analyses with posteriori conceptualizations, self-compassion was described as involving an awareness of the need to care for oneself and indeed to be an active agent in this sense, [52] whereas adopting self-kindness towards one’s psychological state and physical body were characterized by acceptance, and the active distillation of negative self-talk and self-criticism [10, 47]:

*“The course taught me so much more about myself & helped me accept my experience” *[47]

### Use of compassion in the care of PwMS

*The use of compassion in the care of PwMS was discussed both in terms of relating to oneself (self-compassion) and in relation to others.* Self-compassion was often used during mindfulness training to allow PwMS to replace prior tendencies towards self-judgment and criticism. For example, in a mixed-methods study from Europe, PwMS reported that mindfulness training aided them in developing a sense of compassion and self-directed kindness, rather than feelings of “*guilt*” and patterns of “*negative self-talk*” that they experienced previously [10]. One participant reported that she could now, *“actually do things on my own and be happy with my own company”* rather than seeking comfort and assurance from others [10, 50]. In a qualitative study from Asia, mindfulness was evoked by PwMS as being a component of self-compassion, in which one could actively replace ruminating on the “*negative impacts*” of MS with “*self-construction and turning the bad feeling into good ones”* where self-kindness included “*not blaming self because of the disease*” [52]. In another mixed-methods study from Europe, PwMS reported that mindfulness training allowed them to learn more about themselves and helped them to “*accept their experience*” living with illness [47]. Notably, PwMS reported initial difficulties channeling a sense of self-compassion during somatically focused mindfulness practices but that this “*changed over time”* as individuals eventually felt more “*accepting*” and “*self-compassionate*” towards themselves. Another mixed-methods study from Scotland, also reported that PwMS found being attentive to bodily sensations increased awareness of MS symptoms, but that eventually “*this initial discomfort with coming face-to-face with their MS changed over time, into a more accepting and self-compassionate approach”* [50]. In one case, a participant recounted how he was able to replace a previously automatic anger response to painful muscle spasms with equanimity instead:

*“An awful lot of MS people, we get really bad sort of spastic muscle spasms and I used to get so angry . . . you know sort of like shouting and swearing and things because there is nothing you can do except wait until it goes, but I learned to be calmer about the episodes, more gentle about it and that really worked very well and that still works”* [50].

A compassionate approach towards one’s experience was also used by PwMS in response to impaired mobility, evoking the idea that for PwMS there may at times be a somatic or functional trigger to practice self-compassion. For example, PwMS reported that while mobility difficulties previously led to negative self-talk and self-blame, such moments now served as a reminder to practice self-compassion [10]

In quantitative analyses, PwMS were reported to present themselves in a more passive and compassionate manner compared to healthy controls, which the authors suggested may predispose PwMS to practice social compliance to avoid conflict [45].

### Outcomes associated with compassion in the care of PwMS

#### Outcomes among PwMS

Across several studies, self-compassion among PwMS varied with self-esteem, mental health diagnoses, QoL, and level of disability. In a mixed-methods study from Europe, participants reported gains in compassion towards oneself since being diagnosed with MS, independent of sex, gender, and ethnicity [49]. In a longitudinal survey study from Australia, factor analysis of the BFiMSS revealed that compassion/empathy was linked to finding benefits within the context of an MS diagnosis, where increased age and time since diagnosis were weakly linked to greater compassion/empathy [44]. A cross-sectional study from Bulgaria indicated that although total self-compassion scores among PwMS did not differ compared with healthy controls, scores on self-compassion subscales did differ according to one’s level of disability. Specifically, those PwMS with the highest level of disability reported greatest self-kindness and lowest self-judgment, while healthy controls reported greatest self-judgment and lowest self-kindness [42].

In other cross-sectional studies, self-compassion was also linked with a variety of physical and mental health outcomes in PwMS. A study from Spain found that after ‘strict confinement’ due to COVID-19, self-compassion among PwMS was significantly and positively correlated to physical role, social function, vitality, and global health, and negatively correlated with global fatigue and cognitive fatigue [46]. A study from Iran found direct, but statistically insignificant correlations between self-esteem and various subscales of self-compassion, such as self-kindness, and self-judgment [40]. A study from Turkey revealed that non-depressed PwMS had higher self-compassion scores and better QoL compared to those with depression [41]. In another study, compassion was positively related to anxiety and inversely related to depression [44]. A study from the USA found that self-compassion was significantly correlated with QoL as well as resilience [43]. In a study from the UK*,* self-compassion had small mediation effects in lessening distress immediately following an MBI, with moderate sized effects at follow-up [10]. In a study from Australia, total scores on the Self-Compassion Scale increased for PwMS following an MBI, while in a study from Scotland, significant, large, and sustained effect sizes were evident for self-compassion immediately following an MBI and 3 months later [39].

#### Outcomes among caregivers of PwMS

A longitudinal study from Australia found that factors of the BFiMSS had external validity, as caregiver ratings of benefit finding were positively, and significantly correlated with BFiMSS factors, including compassion/empathy, and overall score among PwMS [44]. In addition, results from a cross-sectional study from Spain indicated that caregivers of PwMS had ‘high’ compassion scores, and that such scores were not significantly correlated to their mental, physical, global health, or fatigue [46].

#### Outcomes among HCPs of PwMS

Healthcare provider (HCP) compassion for PwMS was not measured directly in any study. However, in a study from Italy, differences in compassion fatigue emerged according to HCP role as well as age. Specifically, neurologists, compared to nurses, had lower compassion satisfaction and an elevated risk of burnout, although both groups reported high levels of compassion fatigue. Younger (< 45 years old) HCPs had lower compassion satisfaction (i.e., derived less fulfillment from vocational role) in caring for PwMS and higher burnout, although both groups had high levels of compassion fatigue [48].

Table 4 provides an overview of the conceptualization, use, and outcomes of compassion in the care of PwMS.

**Table 4:** Conceptualization, use, and outcomes associated with compassion in the care of people with multiple sclerosis

Study	Aims	Methods	Conceptualization	Use	Outcomes
Bogosian et al. [10]	To explore potential treatment mechanisms of mindfulness-based cognitive therapy to decrease distress in people with multiple sclerosis (PwMS) and to explore participant perspectives on this intervention	Mixed methods; RCT and qualitative interviews	A priori definition—self-compassion using Self-Compassion Scale (SCS) “Defined as the desire to ease one's own suffering through offering self-kindness, and non-judgmental understanding”	Mindfulness training helped participants develop self-compassion and self-directed kindness	Self-compassion had small mediatory role in reduction of distress
Chesi et al. [48]	To define the quality of life (QoL) of Italian neurologists and nurses’ caring for PwMS, and to identify signs of compassion fatigue	Mixed methods; ‘quali-quantitative survey’	A priori definition—compassion fatigue, subscale on Professional Quality of Life Scale “Compassion fatigue is a state of physical or psychological distress, consequent to an ongoing process in a demanding relationship with needy individuals”	N/A	Neurologists, compared to nurses, had lower compassion satisfaction (*p* < 0.001) and elevated burnout risk (*p* < 0.001). Young health care professionals had lower compassion satisfaction (*p* < 0.001) and higher burnout risk (*p* = 0.05–0.01)
Dahmardeh et al. [52]	To explore the meaning of self-compassion experienced by PwMS	Qualitative; interviews	A priori definition—self-compassion Self-compassion is “divided into three primary categories (common humanity, mindfulness, and self-kindness) and three subcategories including over-identifying, self-judgment, and feeling isolated”	Mindfulness (as component of SCS) used by PwMS to actively replace ruminating on the “*negative impacts*” of MS with “*self-construction and turning the bad feeling into good ones”* as well as self-kindness in the form of “*not blaming self because of the disease*”	Individual qualitative interviews, i.e., “How do you feel about yourself when you think about or see other people suffering from MS as well?”
Dahmardeh et al. [40]	To determine the correlation between self-esteem and self-compassion in PwMS	Quantitative; cross-sectional survey	A priori definition—self-compassion “Self-compassion is an adaptive way of communicating with oneself when a person becomes aware of incompetence and encounters difficult situations in life, such as interpersonal problems, leaving behind trauma and natural disasters, and chronic diseases and causes a person to have a cautious and non-judgmental view of oneself, including failures and incompetence”	N/A	This study measured self-compassion via an unvalidated questionnaire designed by research team, reporting a statistically insignificant correlation between self-esteem and various subscales of self-compassion, such as self-kindness (*r* = 0.205; *p* = 0.149), self-judgment (*r* = 0.024; *p* = 0.868), common sense of humanity (*r* = 0.111; *p* = 0.437) and mindfulness (*r* = 0.196; *p* = 0.169) among others
Davidescu et al. [45]	To determine which personality traits are common in PwMS compared to controls	Quantitative; survey	Not explicitly defined. Study used the DECAS Inventory Profile, which measures ‘compassionate’ as a component of interaction with people	PwMS were more often passive and compassionate compared to healthy control participants (*p* = 0.154). Study authors link this with tendency for conflict avoidance, poor expression of preferences	N/A
Gedik et al. [41]	To examine the link between health-related QoL and mental health, self-esteem, as well as self-compassion in PwMS	Quantitative; cross-sectional survey	A priori definition—self-compassion, using SCS “Self-compassion is defined as approaching oneself in a compassionate manner, being mindful of negative emotions without over-identifying with them, and seeing one’s own failures as a natural part of being human”	N/A	Non-depressed PwMS had higher SCS scores compared to those with depression *(t *[54] = 3.82, *p* < 0.001). Total health-related Qol was positively correlated with self-compassion (*r* = 0.42, *p* < 0.01)
Giménez-Llort et al. [46]	To explore the physical and psychological impact of the COVID-19 pandemic on the people with MS and caregivers	Quantitative; survey	A priori definition—compassion, via the compassion scale, self-compassion via SCS “The ability to establish sincere and empathetic connections with the suffering of others and to feel the desire to relieve their pain” “Self-compassion is defined as the ability to understand and support oneself in challenging moments, bearing one’s suffering with kindness and warmth as if it were an inward compassionate action and identifying what is needed to face this situation”	N/A	SCS scores among PwMS were medium–high. Correlations between self-compassion in PwMS and social function, vitality, and global health (*p* < 0.01), and physical role (*p* < 0.05) were positive and significant. A negative correlation was found between self-compassion and global fatigue (*p* < 0.01), self-compassion and cognitive fatigue (*p* < 0.05)
Ignatova et al. [42]	To examine the impairment of social cognition and its potential relationship with grade of disability in MS patients	Quantitative; survey	Self-compassion, using SCS, conflated by study authors with ‘empathy’	N/A	Self-judgment highest in healthy controls, lowest among those with greatest disability. Self-kindness highest among those with high disability, and lowest in individuals with low disability (*p* = 0.004)
Lex et al. [49]	To examine socio-emotional aspects of QoL among those with MS	Mixed methods; survey and qualitative interviews	This study used the Benefit Finding in Multiple Sclerosis Scale (BFiMSS)	N/A	Benefits associated with having MS included gains in compassion since having MS **(***M* = 2.19, SD = 0.05), as revealed by mean scores > 2 on the BFiMSS
Nery-Hurwit et al. [43]	To examine the roles of self-compassion and resilience on health-related QoL for PwMS	Quantitative; survey	A priori definition—self-compassion, using SCS “Self-compassion is defined as the desire to ease one's own suffering through offering self-kindness, and non-judgmental understanding.”	In mediation analysis, self-compassion had direct effect on health-related QoL (*β* 0.49, *p* < 0.0001, CI 0.37–0.61)	Self-compassion significantly positively correlated with health-related QoL (*r* = 0.65, *p* < 0.0001) and resilience (*r* = 0.59, *p* < 0.0001)
Pakenham et al. [44]	To investigate the BFiMSS factors and relation between these factors and adjustment outcomes for PwMS	Quantitative; survey	This study used the BFiMSS—factor analysis revealed compassion/empathy as a factor	N/A	Compassion/empathy linked to benefit finding within the context of MS. Increased age [*r* (380) = 0.10, *p* < 0.05] and time since diagnosis [*r* (371) = 0.16, *p* < 0.01] were weakly linked to greater compassion/empathy. Compassion/empathy was positively related to anxiety (*p* = 0.16) and inversely related to depression (*p* = − 0.04)
Simpson et al. [39]	To test the feasibility of mindfulness-based stress reduction (MBSR) intervention for PwMS	RCT	Not defined per se, but used Self-Compassion Scale-short form (SCS-sf)	Participants were taught MBSR, which included compassion practices	Significant large effect size increase in self-compassion (ES 0.80; *p* < 0.01) immediately following MBSR, sustained 3 months later (ES 0.80; *p* < 0.05)
Simpson et al. [50]	To gather feedback from PwMS after completing a MBSR course	Mixed methods; RCT data and qualitative interviews	Not defined explicitly	Self-compassion taught by MBSR instructors as ‘advocating kindness to the body’	Qualitative interviews
Simpson et al. [51]	To study barriers and facilitators of implementing MBSR for PwMS	Mixed methods; RCT data and qualitative interviews	Not defined	MBSR seen as way to help PWMS become more accepting of the condition, altering relationship with unpleasant embodiment experiences	Qualitative interviews
Spitzer et al. [47]	To evaluate a community-based group mindfulness program for PwMS	Mixed methods; pre-post interventional (adapted MBSR/MBCT) study with written qualitative feedback	A priori definition—self-compassion, using SCS “Self-compassion has three components: self-kindness versus self-judgment, common humanity versus isolation, and mindfulness versus over identification.”	PwMS reported that increased self-compassion following mindfulness training allowed them to learn more about themselves and utilize the practice of acceptance	SCS increased significantly following mindfulness training, *t*(18) = 2.25, *p* = 0.03, with sustained effects at 3-months follow-up *t*(18) = 2.34, *p* = 0.03

*N/A* not applicable

## Discussion

### Summary of findings

This scoping review has explored the conceptualization, use, and outcomes associated with compassion in the care of PwMS. Among studies included in this review, compassion was most often conceptualized based on established, a priori definitions, including compassion in relation to oneself and, as is more traditional, towards others. However, posteriori definitions exclusively described self-compassion, conceptualized as involving self-kindness towards one’s psychological and physical being, abandonment of self-criticism, acceptance, and a sense of agency. Quantitative findings suggest that greater compassion may mediate benefit finding, reduced distress, and better QoL among PwMS, that those with the condition become more compassionate through time, and that self-compassion specifically can be increased through training in mindfulness. In terms of outcomes, greater self-compassion among PwMS correlates with less depression and fatigue, better resilience and QoL.

### Comparison to existing literature

#### Conceptualization of compassion

Considerable debate remains as to the construct of compassion and its conceptualization generally [11, 55]. In the context of MS, very little data exists on how PwMS conceptualize compassion *from* others, or how HCPs use compassion in caring for those with the condition. This latter point is a notable finding, in part because more general evidence syntheses suggest that compassion is most often studied in HCPs and much less so among patients [11], but also because PwMS value when HCPs attune to their emotional needs and take steps to address their distress [5, 56]. Indeed, PwMS report how a perceived lack of *empathy* from HCPs weighs heavily in the ‘cost’ they consider when deciding whether or not to seek out help through mainstream health services [57]. This is problematic for many reasons. Firstly, the needs of PwMS are complex and are accentuated at times of greatest stress, such as at diagnosis, during a relapse, or as disability progresses [58–62]. Secondly, many of the symptoms of MS are ‘hidden’ [63] and therefore may go unnoticed by HCPs, perhaps explaining the lack of attention as perceived by PwMS. However, as the empirical definition of compassion depends upon the recognition of suffering in another, it seems crucially important that HCPs know to ask about ‘hidden’ suffering, even if not apparent on superficial review, besides how to respond [with compassion] when hidden suffering does exist. If such needs are not met in mainstream services, PwMS may end up paying out of pocket for alternative treatments [64, 65] where providers may be perceived as having better ‘listening skills’ and demonstrating ‘more care and concern’ [66].

The debate as to the conceptualization of compassion generally notwithstanding, it seems important to better understand what compassion means to PwMS and their HCPs. This type of study has been done previously in palliative care, using a Grounded Theory approach, with findings suggesting distinct and overlapping meanings for compassion between patients and HCPs [67, 68]. Pursuing such an approach would appear beneficial in informing care optimization for PwMS, given the current mismatch reported between what care PwMS want, versus what they receive [5, 56, 57, 62].

#### Use of compassion

In terms of how compassion is *used* in the care of PwMS, findings suggest that self-compassion is an important factor in the adjustment process, where benefit finding plays a part in discovering positive perspectives on life because of, or in spite of, having MS. This fits with the general academic literature on benefit finding, where factor analysis has previously confirmed compassion as an important component in this complex, multifactorial process, along with conceptually similar post-traumatic growth models [69]. However, besides training in MBIs, which addresses compassion broadly and, arguably, indirectly [9], from this review of the literature it seems that compassion is not being used explicitly in the care of PwMS, by either HCPs or caregivers.

Compassion should, in theory, be a core part of HCP treatment of PwMS. However, high levels of compassion fatigue and burnout are reported in key specialties caring routinely for PwMS, including Neurology [70], Rehabilitation Medicine [26], and Primary Care [27], a scenario likely to have been accentuated greatly during the COVID-19 pandemic. Known drivers of compassion fatigue and burnout (time constraints, inadequate staffing, excessive workload, care fragmentation, use of technology, lack of resources, organizational culture) [71] have also been identified as common barriers to the delivery of compassionate care [11]. Compassion fatigue and HCP burnout matter because they are associated with reduced productivity, lower quality patient care, and worse health in HCPs, increased risk of medical errors, increased costs, and lower patient satisfaction [71]. Interventions for compassion fatigue and burnout need to be multifaceted to be effective [72], but arguably should include compassion-based interventions [22, 29, 73, 74]. However, how compassion is defined in this context needs to be considered carefully [75, 76] as the link between the empirical definition of compassion [14, 75] and compassion fatigue, although perhaps intuitive, is scientifically tenuous at best [77].

Only one study in this review addressed the role of compassion among PwMS within the context of the COVID-19 pandemic [46]. Further study in this area may be beneficial, as in the general population, greater perceived compassion from others and towards one’s self are associated with less psychological distress and fears of contracting COVID-19 [78]. Virtual interventions to facilitate increased self-compassion among PwMS may represent an effective way of addressing the surge of psychological distress reported by PwMS during the COVID-19 pandemic [79, 80]. Online MBIs appear to be acceptable to PwMS [81], are effective at reducing stress [82, 83] and improving self-compassion [69], but do not have compassion as the core focus of treatment and compassion-based interventions may be more effective at improving compassion specifically [9].

Nevertheless, mindfulness and compassion in the care of PwMS appear linked, in that MBI training is associated with an increase in levels of self-compassion while course participants also describe a newfound sense of care and concern for an ailing body. Qualitative research findings in this review certainly suggest that MBI training can help PwMS to develop a more compassionate approach toward themselves, and in relation to others, which may be associated with improved interpersonal functioning and social supports, and fits with existing evidence in general that MBI training is an effective way of improving compassion [29], prosociality [84], and interpersonal relationships [54]. However, learning to be compassionate towards oneself and/or others may take time develop and those PwMS who are older and more disabled report being more compassionate in comparison to younger and less disabled counterparts. When viewed as part of a longitudinal adjustment process, this finding may in part be explained by MS being a ‘moving target’ [85, 86]; particularly in the early stages where fluctuations in disease activity, treatment regimens, and distressing symptoms can be pronounced [87, 88], and present the greatest challenges socially [89, 90].

#### Outcomes of compassion

In terms of *outcomes*, the measurement of compassion among PwMS is a relatively nascent area. Quantitative measures most commonly included the Self-Compassion Scale, which is a six-factor scale including dimensions of self-kindness, self-judgment, common humanity, isolation, mindfulness, and over-identification [91]. Although widely validated in the non-MS literature, this measure has not been validated specifically for use among PwMS, which matters because generic well-being outcome measures may fail to capture what matters most to those affected with MS [92]. Furthermore, the lack of empirical validation for PwMS limits interpretation of findings, in that although there may be face validity for the construct of compassion in the context of managing MS, we cannot be sure about the ecological validity and reliability of the measurement scales in this specific population, as this has not been empirically tested.

Lastly, it is important to determine the outcomes of compassion among caregivers of PwMS. Within the literature examining the experiences of caregivers for older adults, and individuals with dementia, compassion is often linked to compassion fatigue [93–96]. Findings from this review suggest that the experiences of caregivers’ of PwMS are not linked to their QoL and well-being [46], but further research is needed to examine specifically the link between compassion and caregiver well-being within the context of caring for a person with MS.

### Strengths and limitations

This scoping review provides a comprehensive examination of the evidence for the conceptualization, use, and outcomes associated with compassion in the care of PwMS. In keeping with the general literature on compassion in healthcare [11], findings from this current review highlight gaps in the evidence base, such as the paucity of RCTs assessing the effectiveness of compassion as an intervention for improving outcomes for PwMS, or even more generally how compassion is used by HCPs in caring for PwMS. A wide range of validated measures of compassion have been applied to PwMS but have not been validated in this population per se. Lastly, participants across included studies were mainly “white”. This raises the question of how compassion in the care of PwMS from diverse ethnic and racial groups is conceptualized, used, and measured, prompting the need for cultural humility and targeted research across more diverse populations. For example, among some cultures compassion may not be viewed positively in a healthcare context [97].

### Implications

Preliminary conceptualizations of compassion among PwMS have been identified in this scoping review, but more qualitative research is needed to better conceive of the meaning of compassion in the care of PwMS, particularly from the perspectives of care partners and HCPs. Once a clear conceptualization is made, then use and outcomes may be more closely scrutinized. Lastly, there is a need to test compassion-based interventions within the context of MS, as most of the synthesized literature reports on MBIs, in which compassion is only a component part [9]. HCPs may wish to consider how to acquire skills in compassion for them to use in their clinical practice, besides how to train PwMS in self-compassion, which seems particularly promising. Recent systematic review evidence on the nature of compassion education reported a large variety of ‘humanities-based reflective practices’ to facilitate learning about compassion in the context of healthcare [22]. Training practices included reflexive writing, visual analysis of images, watching movies and listening to music [22]. Additional studies indicate that role play and mindfulness may facilitate the cultivation of compassion [73, 98]

## Conclusion

A nascent literature exists on the conceptualization, use, and outcomes associated with compassion in the care of PwMS. Further research is required to better understand what compassion means to PwMS and those caring for them. However, self-compassion can be cultivated among PwMS and may be helpful for managing unpleasant somatic symptoms and in benefit finding. Impact on other health outcomes is less clear. The use of compassion by health care providers in the care of PwMS is unstudied.
